# Validity and reliability of the Depression Information Needs Scale among the Iranian general population

**DOI:** 10.3389/fpsyt.2024.1388447

**Published:** 2024-09-03

**Authors:** Alireza Jafari, Fatemehzahra Naddafi, Mahbobeh Nejatian, Elham Charoghchian Khorasani, Hadi Tehrani

**Affiliations:** ^1^ Department of Health Education and Health Promotion, School of Health, Social Development and Health Promotion Research Center, Gonabad University of Medical Sciences, Gonabad, Iran; ^2^ Student Research Committee, Gonabad University of Medical Sciences, Gonabad, Iran; ^3^ Social Determinants of Health Research Center, Gonabad University of Medical Sciences, Gonabad, Iran; ^4^ Social Determinants of Health Research Center, Mashhad University of Medical Sciences, Mashhad, Iran; ^5^ Department of Health Education and Health Promotion, School of Health, Mashhad University of Medical Sciences, Mashhad, Iran

**Keywords:** psychometric, validity, reliability, depression, translation

## Abstract

**Introduction:**

The prevalence of depression in the community is high. Therefore, it is necessary to examine the information needs on depression in the community. This cross-sectional study aimed to translate and evaluate the psychometric properties of the Depression Information Needs Scale (DINS) among the general population.

**Methods:**

The translation and assessment of the validity and reliability of the DINS were conducted from February 2022 to May 2023 in Gonabad, Iran. The inclusion criteria in this study were individuals 18 years or older, those living in Gonabad for 1 year or more, and participants who provided written informed consent. Sample sizes of 546 and 629 were used for the exploratory factor analysis (EFA) and confirmatory factor analysis (CFA), respectively. The reliability of the DINS was examined using three methods: McDonald’s omega coefficient, test–retest reliability, and Cronbach’s alpha coefficient.

**Results:**

Most participants were women, had a bachelor’s degree, and were married. The values of 0.959 for scale content validity index averaging (S-CVI/Ave) and 0.817 for content validity ratio (CVR) were calculated. In the EFA section, four factors with eigenvalues greater than 1 were extracted and explained 63.861% of the variance. Only two items were not placed in related or acceptable factors and were deleted. Finally, based on the results of the goodness-of-fit indexes (e.g., RMSEA = 0.074, CFI = 0.944, NFI = 0.930, and GFI = 0.911), the scale was approved with 18 items and 4 factors: lived experience (4 items), general (facts about depression) (6 items), research and policies (4 items), and specific treatments (4 items). For all the DINS items, the McDonald’s omega coefficient, Cronbach’s alpha coefficient, and Intraclass Correlation Coefficient (ICC) were 0.953, 0.950, and 0.957, respectively.

**Conclusion:**

The Persian version of the DINS was validated with 18 items and 4 factors, and this scale can be used to assess depression information needs in the general public and specific groups.

## Introduction

Depression is one of the most common mental illnesses, affecting approximately 280 million people around the world. Depression is one of the main causes of suicide, the major cause of disability, and the second leading cause of death among individuals 15–29 years of age (1–4). A systematic review study in Iran has shown that 52% of Iranian elderly (5) and 42.3% of Iranian children suffer from depression (6). In another systematic review, 2.3% of men and 4.8% of Iranian women had major depression disorder (7).

Depression is associated with loss of pleasure, sadness, guilt, weak focus, appetite disorders, sleep disorders, and fatigue (8). Studies have shown that depression is associated with decreased social performance and quality of life and increased risks of chronic diseases such as hypertension, diabetes, coronary artery disease, and even death (3, 9, 10). Depression prevention can reduce the burden of depression disorders, the occurrence of new episodes of major depression disorder, and costs (11). One of the initial depression prevention strategies is education and public awareness programs (12, 13), which in turn strengthen individual empowerment, improve self-management, improve treatment, facilitate decision-making, and ultimately improve mental health (14).

Unfortunately, in mental health educational resources, individuals’ educational needs in terms of mental health are rarely considered, and it is necessary to conduct needs assessment before any educational intervention is conducted to ensure its effectiveness (14). Assessment of information needs identifies the gap between current and desired situations. Needs assessment also facilitates community participation in programs, creating a basis for program analysis and preventing resource loss (15, 16). It will not be possible to understand and recognize the needs for depression information without a valid and reliable tool (14).

A study by Ghadirian et al. in 2017 in a population of people aged 18 to 68 living in Tehran showed that approximately half of the participants were not only unable to identify the signs and symptoms of depression but also did not intend to seek help (17). These findings indicate that Iranian adults have unmet informational needs related to depression. In another study of medical students in Iran, it was found that approximately 64.4% of the students were not able to recognize the signs and symptoms of depression, which highlights the depth of this information gap because it is expected that medical students will have more knowledge in this field (18). The results of another study conducted in 2020 on the general Iranian population revealed an inadequacy of depression literacy in most participants (19). These studies collectively highlight the unmet informational needs of depression and the necessity of comprehensive needs assessment and the implementation of targeted educational interventions. Furthermore, these studies raise the possibility that previous education on depression was not based on individuals’ real information needs and was not successful in promoting depression literacy. Therefore, untargeted depression education could only result in a waste of public funds.

One of the best tools for examining depression information needs is the Depression Information Needs Scale (DINS), which was designed by Griffiths and Crisp. The questionnaire has 20 questions and 4 subscales of lived experience, general (facts about depression), specific treatments, and research and policies (14). Based on the our search, there was not a suitable instrument can assess the unmet information needs of the Iranian population regards to depression. Given the importance of depression and the information needs for this disease, this cross-sectional study aimed to translate the DINS into the Persian language and evaluate its psychometric properties among the Iranian general population.

## Methods

This cross-sectional study was conducted in Iran involving 1,175 patients referred to health centers from February 2022 to May 2023.

### Sample size

Based on the previous studies by Tabatchnick and Williams, the recommended sample size for this type of study would be 500 or more (20, 21). Different samples need to be performed for exploratory factor analysis (EFA) and confirmatory factor analysis (CFA) (22). In this psychometric study, EFA and CFA were assessed using different sample sizes: 546 for EFA and 629 for CFA.

### Sampling

Stratified random sampling was applied in this study. Considering that the target population of this study involved adults aged 18 years and older, community health centers were selected as the most suitable locations for sampling. In these centers, all members of society in any age group, including children, teenagers, adults, and the elderly, have electronic health records, and in this way, they receive health services either by telephone or in person. As a result, these centers provided researchers with the most comprehensive sampling frameworks; thus, they were selected as our sampling settings. After selecting all Gonabad health centers (*n* = 3) as strata, simple random sampling was performed according to the sampling framework and population ratio of each center. The inclusion criteria in this study were individuals aged 18 years or older, those living in Gonabad for more than 1 year, and participants who provided written informed consent to participate in this study. The only exclusion criterion in this study was an incomplete response to the questionnaire (questionnaire with more than 20 % missing data).

### Instruments

The DINS, developed by Griffiths and Crisp, is one of the instruments used for assessing depression information needs, with 20 items and 4 subscales: lived experience (with 5 items), general (facts about depression) (with 7 items), research and policies (with 4 items), and specific treatments (with 4 items) (14). The items were scored using a five-point Likert scale, with 0 indicating completely disagree and 4 indicating completely agree. The minimum score is 0 and the maximum is 80, and a high score indicates that people need more information on depression (14). The validity results of this tool provided four factors, and the DINS had good reliability (Cronbach’s alpha coefficients of the general subscales, specific treatment, research and policies, and lived experience were 0.95, 0.92, 0.91, and 0.96, respectively) (14).

### Translation and cultural adaptation

At first required written consent was obtained from Dr. Kathy Griffiths. After that, the DINS was translated according to the translation and cultural adaptation guidelines (23). First, two translators were recruited to translate the DINS from the original language into the target language (English to Persian). In the second stage, the research team combined the two translations and discussed possible differences. In the third step, two translators translated the combined version of the second step from the target language to the original language (Persian to English). In the fourth step, the two English translations were merged, and the merged English version was translated into Persian in the final step and used to evaluate the validity and reliability of the DINS.

### Validity

To assess the content and face validity, the tool was sent to 11 experts (psychology professionals and specialists in health education and promotion), and their comments were used in the questionnaire. Also, qualitative face validity of the items of DINS were evaluated by 11 people in target group. Quantitative content validity was assessed using content validity ratio (CVR) and scale content validity index averaging (S-CVI/Ave). In the S-CVI/Ave section, each question of the DINS was assessed in terms of relevance (24), and a value >0.9 is acceptable for the S-CVI/Ave section (25), and a value >0.59 is acceptable for CVR (26). The modified kappa value was also calculated for each DINS item. A value from 0.40 to 0.59 is fair, a value from 0.60 to 0.74 is good, and a value >0.74 is excellent (24, 27).

### EFA

Exploratory factor analysis was used to identify extractable factors. To this end, a factor loading ≥0.4, a scree map, and a maximum of 25 rotation repetitions were considered (28, 29). To perform the EFA, sample size sufficiency was assessed by Bartlett’s Test of Sphericity (BTS) and Kaiser–Meyer–Olkin (KMO) (30, 31). This section was implemented using SPSS v24.

### CFA

Factors extracted in EFA were assessed in CFA using AMOS v24. In this section, to prepare the data for analysis, the Mahalanobis test was used to find the outlier data. To check the data normality, skewness and kurtosis were used. After preparing the data, factors extracted were evaluated by goodness-of-fit indexes such as the comparative fit index (CFI), Tucker–Lewis index (TLI), parsimonious normed fit index (PNFI), relative fit index (RFI), chi-square (*χ* ^2^), degree of freedom (*df*), parsimony comparative fit index (PCFI), normed fit index (NFI), incremental fit index (IFI), root mean square error of approximation (RMSEA), and goodness-of-fit index (GFI) (32–34). Based on the literature, the acceptable value for each goodness-of-fit index is IFI >0.9, TLI >0.9, PNFI >0.5, RMSEA <0.08, GFI >0.9, NFI >0.9, CFI >0.9, *χ* ^2^/*df <*5, PCFI >0.5, and RFI >0.9 (32–35).

### Reliability

The reliability of the DINS was examined using three methods: McDonald’s omega coefficient, test–retest reliability, and Cronbach’s alpha. In test–retest reliability, the Intraclass Correlation Coefficient (ICC) was calculated. To check the reliability, 37 participants were studied, and the questionnaire was completed two times; the second time was 1 month after the first time. To evaluate reliability, two software programs (JASP v0.11.1.0 and SPSS v24) were used. Regarding Cronbach’s alpha, 0.6 ≤ *α* ≤ 0.7 is acceptable and *α* ≥0.8 is very good (36). ICC >0.9 is excellent, 0.75 ≤ ICC ≤ 0.9 is good, 0.5 ≤ ICC ≤ 0.75 is moderate, and ICC <0.5 is poor (37). In general, a reliability coefficient greater than 0.70 is acceptable (38).

## Results

### Demographic characteristics

In EFA, 51.1% (*n* = 279) were male, and in CFA, 53.7% (*n* = 338) were female (**Table 1**). In the EFA and CFA, the mean (SD) age of the participants was 33.91 (13.12) and 33.17 (13), respectively.

**Table 1:** Frequency distribution of demographic characteristics.

Variables		EFA (*n* = 546)		CFA (*n* = 629)	
		*n*	%	*n*	%
**Sex**	Female	267	48.9	338	53.7
	Male	279	51.1	291	46.3
**Occupation**	Self-employed	78	14.3	96	15.3
	Employed	140	25.6	166	26.4
	Retired	34	6.2	28	4.5
	Housewife	66	12.1	64	10.1
	Laborer	22	4.1	28	4.4
	University student	206	37.7	247	39.3
**Marital status**	Married	319	58.4	352	56
	Single	227	41.6	266	44
**Education level**	Elementary school	10	1.8	14	2.3
	Middle school	19	3.4	18	2.8
	High school	26	4.8	19	3
	Diploma	157	28.8	180	28.6
	Associate degree	74	13.6	96	15.3
	Bachelor’s degree	196	35.9	220	35
	Master’s degree or high degree	64	11.7	82	13

### Face and content validity

After the validity assessment by 11 experts, two items in qualitative face validity and three items in qualitative content validity were modified (using simple and appropriate Persian words), respectively. In terms of quantitative content validity, values of 0.959 for S-CVI/Ave and 0.817 for CVR were calculated. The modified kappa for the 18 items was excellent, and the value for all items was 0.926 (**Table 2**).

**Table 2:** The I-CVI and modified kappa for each item of the DINS.

Items	Number of agreement	I-CVI (item-level content validity index)	Pc (probability of chance agreement)	*K* (modified kappa)	Evaluation
1	11	1	0.000488281	1	Excellent
2	10	0.909090909	0.005371094	0.908599991	Excellent
3	11	1	0.000488281	1	Excellent
4	11	1	0.000488281	1	Excellent
5	10	0.909090909	0.005371094	0.908599991	Excellent
6	11	1	0.000488281	1	Excellent
7	11	1	0.000488281	1	Excellent
8	11	1	0.000488281	1	Excellent
9	11	1	0.000488281	1	Excellent
10	10	0.909090909	0.005371094	0.908599991	Excellent
11	10	0.909090909	0.005371094	0.908599991	Excellent
12	11	1	0.000488281	1	Excellent
13	10	0.909090909	0.005371094	0.908599991	Excellent
14	8	0.727272727	0.080566406	0.703374692	Good
15	7	0.636363636	0.161132813	0.566514975	Fair
16	9	0.818181818	0.026855469	0.813164257	Excellent
17	11	1	0.000488281	1	Excellent
18	11	1	0.000488281	1	Excellent
19	10	0.909090909	0.005371094	0.908599991	Excellent
20	11	1	0.000488281	1	Excellent

### EFA section

**Figure 1:**
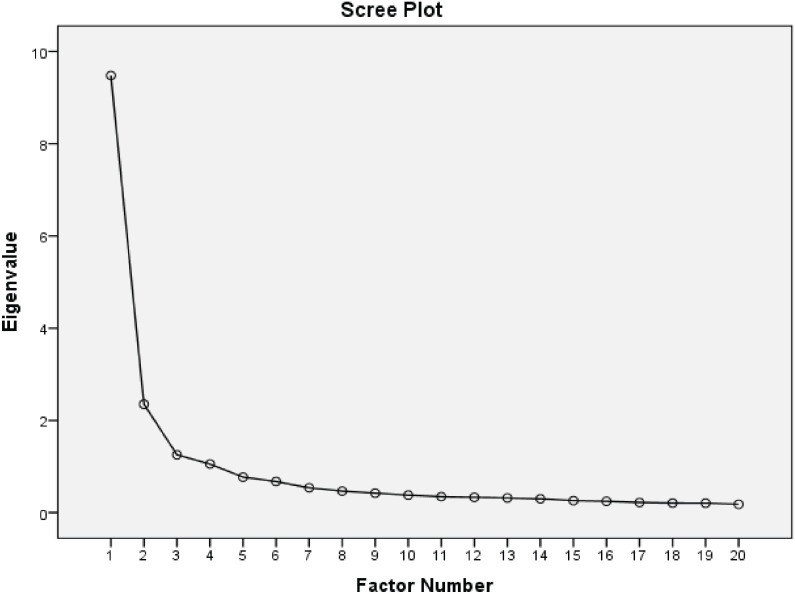
Scree plot of the factor analysis of the Persian version of the DINS.

There were no missing data in this section. The results of the KMO (0.936) and BTS (*χ* ^2^ = 7,603.659, *df* = 190, *P* < 0.001) showed that the samples were sufficient for EFA. In this section, four factors with eigenvalues greater than 1 were extracted and explained 63.861% of the variance (**Table 3**, **Figure 1**). The results of **Table 4** show the place of each item in extracted factors and based on the original scale; only two items did not place in related and acceptable factors. Item D7 (“How I can help someone who is depressed”) moved from the general (facts about depression) factor to the lived experience factor, and item D12 (“People’s experiences of which treatments work for their depression”) moved from the lived experience factor to the specific treatments factor. After checking these results, two items, i.e., D7 and D12, were deleted (**Table 4**).

**Table 3:** The four-factor structure of the Persian version of the DINS.

Total variance explained									
Factor	Initial eigenvalues			Extraction sums of squared loadings			Rotation sums of squared loadings		
	Total	% of variance	Cumulative %	Total	% of variance	Cumulative %	Total	% of variance	Cumulative %
1	9.480	47.398	47.398	9.112	45.559	45.559	3.757	18.784	18.784
2	2.351	11.756	59.154	2.003	10.014	55.572	3.264	16.318	35.102
3	1.255	6.273	65.427	0.948	4.741	60.313	3.262	16.309	51.411
4	1.054	5.268	70.695	0.710	3.548	63.861	2.490	12.449	63.861
5	0.769	3.846	74.541						
6	0.674	3.371	77.913						
7	0.539	2.693	80.606						
8	0.466	2.331	82.937						
9	0.422	2.108	85.045						
10	0.379	1.895	86.940						
11	0.346	1.729	88.669						
12	0.331	1.657	90.326						
13	0.318	1.591	91.917						
14	0.298	1.492	93.409						
15	0.260	1.298	94.708						
16	0.246	1.230	95.938						
17	0.220	1.099	97.037						
18	0.207	1.036	98.072						
19	0.205	1.025	99.098						
20	0.180	0.902	100.000						

Extraction method: maximum likelihood.

**Table 4:** Rotated factor matrix of the Persian version of the DINS.

Rotated factor matrix^a^				
Items	Factor			
	1	2	3	4
D2	0.794	0.230	0.166	0.162
D3	0.760	0.245	0.202	0.143
D1	0.731	0.172	0.160	0.095
D4	0.688	0.331	0.209	0.171
D5	0.573	0.358	0.137	0.184
D6	0.525	0.401	0.165	0.190
D9	0.286	0.810	0.232	0.131
D10	0.256	0.744	0.141	0.186
D11	0.287	0.700	0.097	0.219
D8	0.358	0.682	0.250	0.076
D7	0.454	0.484	0.306	0.120
D18	0.170	0.203	0.792	0.300
D19	0.232	0.137	0.750	0.200
D20	0.219	0.132	0.702	0.302
D17	0.160	0.250	0.699	0.311
D12	0.310	0.272	0.398	0.381
D14	0.162	0.171	0.323	0.786
D15	0.104	0.103	0.251	0.668
D13	0.221	0.174	0.282	0.651
D16	0.181	0.198	0.512	0.513

Extraction method: maximum likelihood; rotation method: varimax with Kaiser normalization.

a Rotation converged in six iterations.

### CFA section

**Figure 2:**
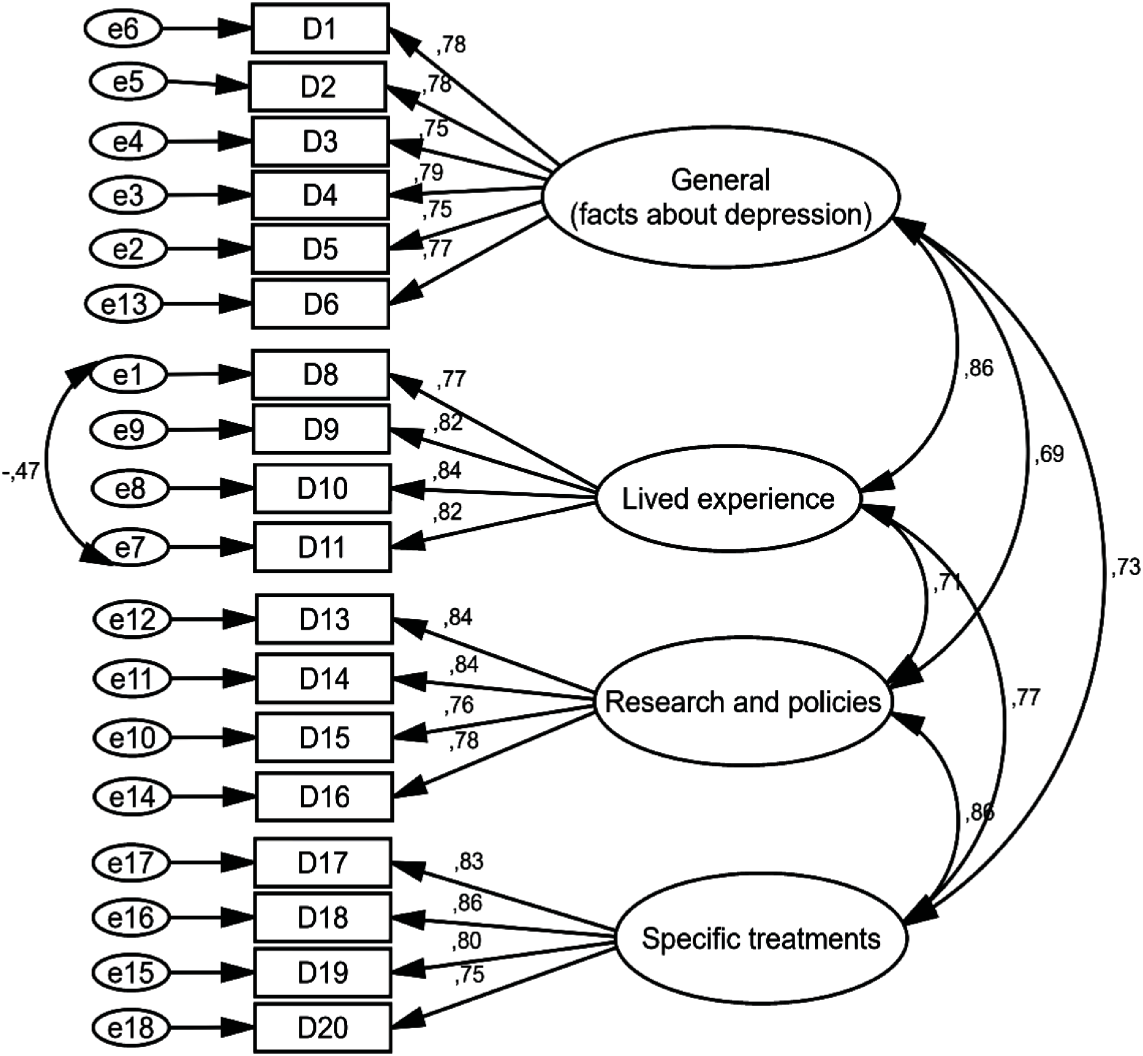
Standardized parameter estimates for the factor structure of the DINS.

In this section, data were normal and there were no missing data. After that, four extracted factors with 18 items were evaluated by CFA. The results of **Table 5** show that the factor loading of all items was greater than 0.7 (**Table 5**, **Figure 2**). Based on the results of **Table 6**, the model fit indicators (such as RMSEA = 0.074, NFI = 0.930) indicated that the final model was acceptable with 18 items and 4 factors: lived experience (4 items), general (facts about depression) (6 items), research and policies (4 items), and specific treatments (4 items) (**Table 6**). The final Persian version of the DINS was uploaded as **Supplementary Material** (**Appendix 1**).

**Table 5:** Factor loadings of the Persian version of the DINS.

Subscales	Items	Factor loadings
**General (facts about depression)**	D1: The symptoms of depression and how to tell if someone is depressed	0.776
	D2: The causes of depression and who is most at risk of depression	0.784
	D3: The course of depression (how long it lasts and if and how it recurs)	0.749
	D4: The treatments that work for depression	0.790
	D5: How common depression is in the community	0.753
	D6: Which professionals and groups can help someone who is depressed	0.770
	D7: *How I can help someone who is depressed*	Deleted*
**Lived experience**	D8: People’s personal stories about coping with depression during the initial stages of an episode of depression	0.770
	D9: People’s personal stories about coping during the recovery phase of depression	0.815
	D10: People’s personal stories about how it feels to be depressed	0.839
	D11: People’s personal stories about the attitudes of others to their depression	0.817
	D12: *People’s experiences of which treatments work for their depression*	Deleted*
**Research and policies**	D13: Workplace depression policies	0.838
	D14: Government policies and strategies for combating depression	0.838
	D15: Funding of research on depression	0.756
	D16: Recent research findings about depression	0.777
**Specific treatments**	D17: The side effects of antidepressants and how to cope with them	0.826
	D18: Which psychological treatments work for depression	0.864
	D19: Which prescription medications work for depression	0.796
	D 20: Which alternative and lifestyle treatments work for depression	0.747

*Moved to the unrelated factor in the EFA.

**Table 6:** The model fit indicators of the Persian version of the DINS.

Goodness-of-fit indices	Confirmatory factor analysis	Acceptable value
***χ* ^2^**	570.367	–
***df***	128	–
***χ* ^2^/*df***	4.456	<5
***P*-value**	0.000	*P* > 0.05
**IFI**	0.945	>0.9
**GFI**	0.911	>0.9
**RMSEA**	0.074	<0.08
**CFI**	0.944	>0.9
**PNFI**	0.778	>0.5
**PCFI**	0.790	>0.5
**NFI**	0.930	>0.9
**RFI**	0.916	>0.9
**TLI**	0.933	>0.9

### Reliability section

**Table 7** shows the scale reliability results. For all DINS items, the McDonald’s omega coefficient, Cronbach’s alpha coefficient, and ICC were 0.953, 0.950, and 0.957, respectively. The reliability of each factor is presented in **Table 7**.

**Table 7:** Reliability and descriptive statistics of the Persian version of the DINS.

Subscales	Item	Range	Cronbach’s alpha coefficients	McDonald’s omega coefficients	Test–retest			*P*-value
					Intraclass correlation coefficient (ICC)	95% Confidence interval		
						Lower bound	Upper bound	
General (facts about depression)	6	0–24	0.916	0.921	0.962	0.926	0.980	<0.001
Lived experience	4	0–16	0.943	0.945	0.947	0.897	0.973	<0.001
Research and policies	4	0–16	0.922	0.925	0.939	0.882	0.696	<0.001
Specific treatments	4	0–16	0.966	0.969	0.960	0.922	0.979	<0.001
Total DINS	18	0–72	0.950	0.953	0.957	0.917	0.978	<0.001

## Discussion

This study aimed to evaluate the psychometric properties of DINS. In general, the Persian version of the DINS demonstrated robust psychometric properties.

Similar to the original DINS and according to the EFA results, the Persian version of the DINS also yielded four factors (general, lived experience, research and policies, and specific treatment) with specific values greater than 1 capable of predicting 63.861% of the variance. According to the EFA results, only two items were not in the relevant and acceptable factors, and 18 items were placed in the four main subscriptions. In addition, these four factors were examined in the CFA stage, and all questions in the questionnaire were confirmed.

In this study, the first factor was “general (facts about depression),” which was confirmed with six questions. This factor relates to the general facts of depression, including symptoms, causes, periods, treatment, outbreaks, and sources, and emphasizes that community members with depression have information needs regarding the general factors of the disease that must be properly available to them. The results of a study by Prins on perceived needs to take care of mental health and depression in the Netherlands showed that information needs were not provided by one-third of the participants about their illness or were provided at a very small amount (39). It is difficult to manage a disease when individuals with depression are not sufficiently well informed about their illness (40). On the other hand, numerous studies have shown that when people obtain accurate information about their illness, they are more likely to follow treatment, and unmet information needs can lead to delayed or inadequate treatment, poor adherence, increased stigma, and a higher risk of relapse or recurrence (41, 42). Therefore, a tool that can assess the depression information needs of individuals is necessary for conducting interventions.

According to the results of this study, the second factor to be approved was “lived experience,” which was confirmed by four questions. The lived experience factor refers to the personal experiences of people with depression regarding coping strategies during the acute period to improve their depression, their treatments, and others’ attitudes toward depression. In the study by Jorm et al., it was mentioned that the beliefs of people with experience of depression about mental disorders differ from those of health professionals. Therefore, patients with depression may have very different opinions from physicians regarding what interventions are useful (43). As a result, the experiences of depression patients can be part of the important information needs for the general people. Some studies have emphasized the critical role of lived experience perspectives in understanding and addressing the unique information needs of people with depression (2, 44). Therefore, incorporating these insights can lead to more impactful, person-centered resources and interventions to support those affected by this condition.

The third factor that was approved was “research and policies,” which was confirmed with four questions. The focus of this factor is on workplace policies, government policies, financing research, and research-related depression results. By understanding the specific areas where information is lacking and implementing policies that enhance access to this information, stakeholders can better support individuals struggling with depression. Patients’ information about the results of research on depression can help them better understand the disease and its effective treatments (45). Policies can also increase confidence among patients with depression in accessing the required care services. Therefore, awareness of research findings and policies is an important information requirement for people with mental disorders (46). Griffiths demonstrated that providing information about workplace laws and government policies to individuals suffering from depression, along with updates on the latest research findings in the field, significantly improved disease management and motivated patients to adhere to their treatment plans (14). Numerous studies underscore the critical role of research and policies in understanding, prioritizing, and addressing the multifaceted information needs of people with depression (2, 47). Therefore, by using an appropriate tool that can measure this factor, more comprehensive and effective informational resources and interventions can be developed to support people affected by depression.

According to the results of this study, the fourth factor that was approved was “specific treatments,” which was confirmed by four questions. This factor refers to information about specific treatments for depression and the side effects of treatments and psychological treatments. The results of a qualitative study by Fossey et al. showed that patients with mild to moderate mental disorders lack information regarding diagnosis and treatment options (48). Bowskill et al. also reported patient dissatisfaction with the information provided by health professionals for mental disorder therapies, including the nature, treatment period, and prescribed medications (49). In general, targeted information is important not only for patients with mental disorders but also for community members because mental health literacy is likely to help people diagnose the primary symptoms of mental illness and seek mental health services in the early stages of the disease (2). Various studies have emphasized the vital role of providing comprehensive and tailored information about specific treatment options to meet the needs of people with depression (14, 50). By prioritizing this aspect, healthcare providers and information resources can support depressed people in making informed choices about their care and participating more actively in the treatment process.

### Strengths and limitations

The large sample size of this study and the fact that it was conducted in the public community were the strengths of the study, and the DINS is useful for different target groups. The use of a self-report questionnaire may introduce cognitive biases, which is a limitation of this research. Another limitation of this study was that it was conducted in a single region and city, which may limit the generalizability of the findings to a broader population of people with depression.

## Conclusion

The Persian version of the DINS is of crucial importance at this juncture given the shift of focus and huge emphasis on mental health worldwide, including Iran. It is hoped that this scale will improve the quality of educational interventions related to depression and address the knowledge gap among the Iranian public. This scale can be used to evaluate the information needs of patients with depression, as well as the effect of various interventions to improve information. The scale can also serve as a basis for developing clinical guidelines for providing appropriate and targeted information to consumers with mental health. However, further research is required to confirm the validity and reliability of the DINS in different subgroups. The replication of the study in other regions, cities, or countries is also suggested to evaluate the transferability of the findings.

## Data Availability

The original contributions presented in the study are included in the article/**Supplementary Materials**. Further inquiries can be directed to the corresponding author.
