# Supplementary material for: Validity and reliability of the Depression Information Needs Scale among the Iranian general population
Source: Front Psychiatry. 2024 Sep 3;15:1388447. doi: 10.3389/fpsyt.2024.1388447 (PMC11406337; doi:10.3389/fpsyt.2024.1388447)
Supplement: Supplementary file 1 [file DataSheet1.zip › Data sheet 1/Appendix file 1-Table s1.docx]

**Appendix file 1**

**Table S1:** **The final Persian version of depression information needs scale (DINS) with 18 items and four subscales**

| **سوالات** | **کاملا مخالفم** | **مخالفم** | **نه مخالف نه موافق** | **موافقم** | **کاملا موافقم** |
| --- | --- | --- | --- | --- | --- |
| 1. من نیاز به اطلاعاتی در مورد علائم افسردگی و تشخیص افسردگی در دیگران دارم | **General (facts about depression)** | | | | |
| 1. من نیاز دارم که از علل افسردگی و اینکه چه کسی بیشتر در معرض خطر افسردگی است اطلاع داشته باشم |  |  |  |  |  |
| 1. من نیاز به اطلاعاتی در مورد طول دوره بیماری افسردگی و ظهور مجدد علائم بیماری (بازگشت بیماری) دارم |  |  |  |  |  |
| 1. من نیاز به اطلاعاتی در مورد درمانهایی که برای افسردگی استفاده می شوند، دارم |  |  |  |  |  |
| 1. من نیاز به اطلاعاتی در مورد میزان شیوع افسردگی در جامعه دارم |  |  |  |  |  |
| 1. من در مورد اینکه کدام متخصصان و گروهها می توانند به کسی که افسرده است کمک کنند، نیاز به اطلاعات دارم |  |  |  |  |  |
| 1. من نیاز دارم که از تجربه های شخصی افراد در مورد مقابله با افسردگی در مراحل اولیه بیماری مطلع شوم | **Lived experience** | | | | |
| 1. من نیاز دارم که از تجربه های شخصی افراد در مورد مقابله آنها در مرحله بهبودی افسردگی مطلع شوم |  |  |  |  |  |
| 1. من نیاز دارم که از تجربه های شخصی افراد در مورد احساس افسردگی آنها مطلع شوم |  |  |  |  |  |
| 1. من نیاز دارم که از تجربه های شخصی افراد در مورد نگرش دیگران به افسردگی آنها، مطلع شوم |  |  |  |  |  |
| 1. من نیاز به اطلاعاتی در مورد سیاست گذاری ها و استراتژی های پیشگیری از افسردگی در محل کار دارم | **Research and policies** | | | | |
| 1. من نیاز به اطلاعاتی در مورد سیاست گذاری ها و استراتژی های دولت برای مبارزه با افسردگی دارم |  |  |  |  |  |
| 1. من نیاز به اطلاعاتی در مورد مقدار بودجه تعیین شده برای پژوهش در زمینه افسردگی دارم |  |  |  |  |  |
| 1. من نیاز به اطلاعاتی در مورد یافته های تحقیقات اخیر در مورد افسردگی دارم |  |  |  |  |  |
| 1. من نیاز به اطلاعاتی درباره عوارض جانبی داروهای افسردگی و نحوه مقابله با آن ها دارم | **Specific treatments** | | | | |
| 1. من نیاز به اطلاعاتی دارم که بدانم کدام درمانهای روانشناسی برای درمان افسردگی مفید ترند |  |  |  |  |  |
| 1. من نیاز دارم که بدانم کدام داروهای تجویزی برای افسردگی مفید هستند |  |  |  |  |  |
| 1. من نیاز دارم که بدانم کدام روش های درمانی طب جایگزین و شیوه زندگی (مثل ورزش کردن، دارو های گیاهی، طب سوزنی و...) برای درمان افسردگی مفید هستند |  |  |  |  |  |
