# Supplementary material for: Validity and reliability of the Depression Information Needs Scale among the Iranian general population
Source: Front Psychiatry. 2024 Sep 3;15:1388447. doi: 10.3389/fpsyt.2024.1388447 (PMC11406337; doi:10.3389/fpsyt.2024.1388447)
Supplement: Supplementary file 1 [file DataSheet1.zip › Data sheet 1/Appendix file 2-Table s2.docx]

**Appendix file 2**

**Table S2:** **The final version of depression information needs scale (DINS) with 18 items and four subscales**

| **Subscales** | **Items**  ******* ***I need information about…*** | **Strongly disagree** | **Disagree** | **Neither agree nor disagree** | **Agree** | **Strongly agree** |
| --- | --- | --- | --- | --- | --- | --- |
| **General (facts about depression)** | 1. The symptoms of depression and how to tell if someone is depressed |  |  |  |  |  |
|  | 1. The causes of depression and who is most at risk of depression |  |  |  |  |  |
|  | 1. The course of depression (how long it lasts and if and how it recurs) |  |  |  |  |  |
|  | 1. The treatments that work for depression |  |  |  |  |  |
|  | 1. How common depression is in the community |  |  |  |  |  |
|  | 1. Which professionals and groups can help someone who is depressed |  |  |  |  |  |
| **Lived experience** | 1. People’s personal stories about coping with depression during the initial stages of an episode of depression |  |  |  |  |  |
|  | 1. People’s personal stories about coping during the recovery phase of depression |  |  |  |  |  |
|  | 1. People’s personal stories about how it feels to be depressed |  |  |  |  |  |
|  | 1. People’s personal stories about the attitudes of others to their depression |  |  |  |  |  |
| **Research and policies** | 1. Workplace depression policies |  |  |  |  |  |
|  | 1. Government policies and strategies for combating depression |  |  |  |  |  |
|  | 1. Funding of research on depression |  |  |  |  |  |
|  | 1. Recent research findings about depression |  |  |  |  |  |
| **Specific treatments** | 1. The side effects of antidepressants and how to cope with them |  |  |  |  |  |
|  | 1. Which psychological treatments work for depression |  |  |  |  |  |
|  | 1. Which prescription medications work for depression |  |  |  |  |  |
|  | 1. Which alternative and lifestyle treatments work for depression |  |  |  |  |  |
